# Investigation of neglected bacterial pathogens provides molecular and serological evidence of *Bartonella* spp. in patients with acute undifferentiated febrile illness in Villeta, Colombia

**DOI:** 10.1186/s13071-026-07351-y

**Published:** 2026-03-11

**Authors:** Carlos Ramiro Silva-Ramos, J. Manuel Matiz-González, Nicole L. Mendell, C. Alexander Barrero-Rubiano, Álvaro A. Faccini-Martínez, Claudia Cuervo, Peter C. Melby, Patricia V. Aguilar, Miguel M. Cabada, David H. Walker, Marylin Hidalgo

**Affiliations:** 1 Grupo de Enfermedades Infecciosas, Departamento de Microbiología, Facultad de Ciencias, Pontificia Universidad Javeriana, Bogotá, Colombia; 2 Department of Pathology, University of Texas Medical Branch, Galveston, TX USA; 3 Programa de Enfermeria, Facultad de Ciencias de la Salud, Universidad Distrital Francisco José de Caldas, Bogotá, Colombia; 4 Molecular Genetics and Antimicrobial Resistance Unit, Universidad El Bosque, Bogotá, Colombia; 5 Servicio de Infectología, Hospital Militar Central, Bogotá, Colombia; 6 Facultad de Medicina, Universidad Militar Nueva Granada, Bogotá, Colombia; 7 Division of Infectious Diseases, Department of Internal Medicine, University of Texas Medical Branch, Galveston, TX USA; 8 Center for Tropical Diseases, University of Texas Medical Branch, Galveston, TX USA

**Keywords:** *Bartonella*, Febrile patients, Colombia, Molecular detection, Serology, Phylogeny, Neglected pathogens

## Abstract

**Background:**

Acute undifferentiated febrile illness (AUFI) is a challenging clinical condition in tropical regions, caused by a broad range of pathogens. In Villeta municipality, Colombia, data on neglected bacterial causes remain scarce, highlighting the need to expand understanding of the local etiological spectrum. Thus, the aim of the present study was to explore the presence of the neglected pathogens, *Bartonella*, *Borrelia*, and *Coxiella burnetii*, as potential causes of AUFI in Villeta.

**Methods:**

DNA was extracted from whole-blood samples from febrile patients. Quality and purity were assessed spectrophotometrically and by conventional polymerase chain reaction (PCR). *Bartonella*, *Borrelia*, and *C. burnetii* were detected using genus- and species-specific quantitative PCR (qPCR) assays. *Bartonella*-positive samples were further analyzed by multigene PCRs and sequencing for species identification. Anti-*Bartonella* and anti-*C. burnetii* immunoglobulin G (IgG) antibodies were evaluated by indirect immunofluorescence to assess recent or past exposure to these agents.

**Results:**

A total of 41 febrile patients were evaluated. *Bartonella* DNA was detected in 9.8% (4/41) of samples. No *Borrelia* or *C burnetii* DNA was detected. Phylogenetic analysis revealed two distinct clades, although none could be assigned to species level. Serological analysis showed anti-*Bartonella* IgG antibodies in 29.3% (12/41) of cases, with 9.8% (4/41) exhibiting seroconversion. One patient presented both molecular and seroconversion evidence of recent *Bartonella* infection. None of the patients were seropositive for *C. burnetii*.

**Conclusions:**

This study provides the first molecular and serological evidence of *Bartonella* circulation among febrile patients in Villeta, Colombia, revealing genetically distinct lineages and indicating both active and past infections, underscoring its potential role in AUFI.

**Graphical Abstract:**

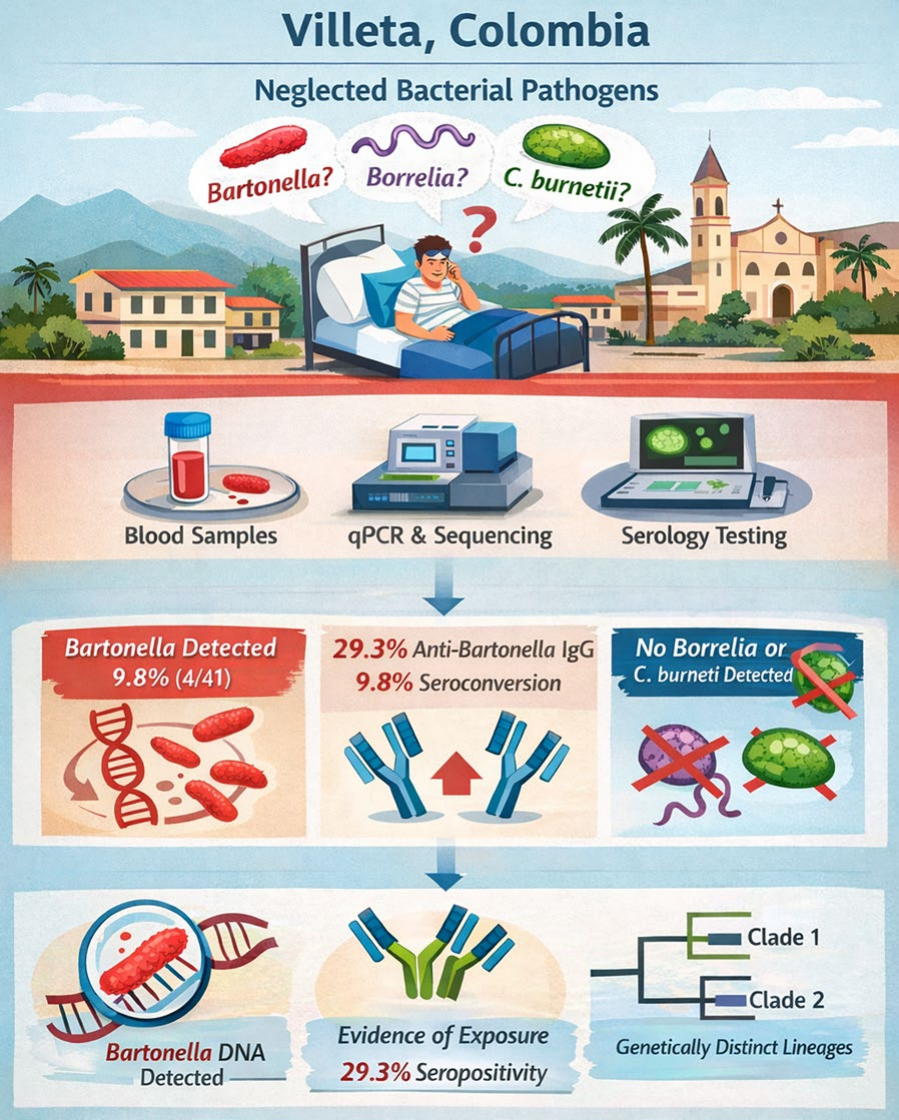

## Background

Acute undifferentiated febrile illness (AUFI) is a syndromic condition characterized by fever and nonspecific symptoms lasting less than 2 weeks, without a clinically localizing source of infection. Diagnosis of AUFI is a great challenge owing to the wide etiological spectrum, mainly in tropical settings [1, 2]. Although AUFI can be self-limiting, severe and fatal cases may occur, requiring prompt recognition to start specific therapy; nevertheless, owing to limited diagnostic resources and the lack of active epidemiological surveillance in tropical endemic regions, it is difficult to establish an etiological diagnosis [1, 3].

In Latin America and the Caribbean, malaria is still a significant cause of AUFI and occurs in at least 21 countries across the region [4]. Currently, arboviral infections due to dengue (DENV), chikungunya, Zika, and Oropouche (OROV) viruses represent the most frequent etiologies [5] as well as other emerging pathogens, including *Leptospira* spp., *Rickettsia* spp., and respiratory viruses [2, 6]. However, the etiological spectrum is broader, and several bacterial agents, particularly *Bartonella* spp., *Borrelia* spp., and *Coxiella burnetii*, remain largely overlooked, representing neglected components of the etiology of AUFI with underestimated importance despite their zoonotic potential and clinical relevance [7].

*Bartonella* is a group of fastidious, intra-erythrocytic, gram-negative, vector-borne bacteria with a broad spectrum of animal reservoir hosts. In addition, it can be transmitted by a wide range of arthropods, including sandflies, lice, fleas, and possibly ticks [8, 9]. *Bartonella bacilliformis*, *B. quintana*, and *B. henselae* are the classical pathogens for humans, causing Carrion’s disease, trench fever, and cat-scratch disease, respectively [9]; however, several emerging and novel *Bartonella* species have been identified as human pathogens [9, 10], expanding the clinical spectrum of infection. Some of these have been linked to febrile illnesses of unknown origin [11].

*Borrelia* spp. are gram-negative spirochetes classified into two main groups: the *Borrelia burgdorferi* sensu lato complex related to Lyme borreliosis and the relapsing fever group (RFG) [12]. The latter comprises a wide range of human pathogenic *Borrelia* species, responsible for relapsing fever illnesses transmitted by human clothing lice or by ticks belonging to the *Argasidae* and *Ixodidae* families [13]. While louse-borne relapsing fever involves humans as sole hosts, tick-borne relapsing fever is a zoonotic, vector-borne, and environmentally persistent disease maintained in nature through several domestic and wild animal species that might act as reservoir hosts and important sources of infection [14].

*Coxiella burnetii*, the causative agent of Q fever, is a zoonotic pathogen mainly transmitted via aerosols, although it can also be transmitted by ticks. This bacterium exhibits two antigenic phase variants. Phase I is a virulent form found in nature. Phase II is a form that arises after repeated in vitro passages, which produces truncated (rough) lipopolysaccharides (LPSs) due to deletions or mutations in LPS-associated genomic regions, leading to structural alterations and a decreased ability to evade the host immune response [15, 16]. An immune response dominated by antibodies to phase I antigens is indicative of a chronic infection, whereas a higher titer against phase II antigens indicates an acute infection [15, 16]. It is resistant to chemical agents and extreme environmental conditions, making it highly infectious and a potential bioterrorism concern [16]. Infection mainly affects rural populations exposed to domestic ruminants, which are the main reservoir hosts of this bacterium [17, 18]. Although Q fever is often a mild self-limiting febrile illness, it can progress to severe forms such as atypical pneumonia, endocarditis, and osteomyelitis, which are the leading causes of death [16, 19].

Villeta municipality is an area with a high incidence of AUFIs. To date, two studies have investigated the etiology of AUFI, implicating DENV, *Leptospira* spp., and spotted fever group (SFG) *Rickettsia* spp. as the most frequent pathogens [20, 21]. Screening for neglected arboviruses such as Mayaro, OROV, and Venezuelan equine encephalitis virus by molecular assays has also been performed, all yielding negative results [21]. However, studies addressing neglected bacterial pathogens remain limited and, in some cases, totally absent. Historical evidence dating back to 1906, suggested the presence of spirochetes morphologically compatible with tick-borne RFG *Borrelia* spp. in blood smears of febrile patients from the region, later reinforced by clinical observations in Villeta and Albán municipalities in 1934 [22, 23]. More recently, a study screened for *Anaplasma spp.* and *C. burnetii* infection by serology, detecting only a single probable case of human anaplasmosis [20]. No attempts have been made to investigate *Bartonella* spp. as a possible cause of febrile illness in Villeta.

The etiology of AUFI is very broad, and regional epidemiological surveillance is essential to identify potential endemic pathogens. Despite previous efforts, important gaps remain in understanding neglected bacterial causes of AUFI in the region. Some pathogens have never been investigated, while for others, only historical evidence is available. Given the lack of data, studying febrile patients represents an initial step within a One Health framework. Therefore, the aim of the present study was to explore the presence of the neglected pathogens, *Bartonella* spp., *Borrelia* spp., and *C. burnetii*, as potential causes of AUFI in Villeta municipality, Colombia, to broaden the current knowledge of the AUFI etiological spectrum in the region.

## Methods

### Study area

Villeta (5°00´46″ N, 74°28´23″ W) is a municipality located at an elevation of 850 m above sea level, in the Gualivá Province, Cundinamarca Department, which is 84 km from Bogotá D.C. It comprises 22 villages distributed over an area of 140 km^2^. The annual mean temperature in the region is 26 °C, with relative humidity ranging between 80% and 97%. Ecotourism and agriculture are the main economic activities in the municipality, with sugarcane cultivation and “panela” production being the most predominant economic activities in rural areas (www.villeta-cundinamarca.gov.co). According to the last National Population and Housing Census conducted by the Departamento Administrativo Nacional de Estadística (DANE) in 2018, Villeta has a total population of 25,957 inhabitants, of which 17,751 reside in the urban area, 743 in suburban settlements, and 7463 in rural areas (https://www.dane.gov.co).

### Sample collection

Active surveillance was conducted between September and December 2021 at the Salazar de Villeta Hospital as part of a larger multisite and multicountry study of AUFI carried out in six regions across four countries, which followed a standardized protocol and data collection system [24]. Male and female patients older than 2 years presenting to the emergency department with a febrile illness of less than 14 days of evolution and without an identifiable source of infection were eligible for inclusion. Fever, defined as a temperature > 37.8 ˚C, had to be documented by the patient or healthcare personnel prior to enrollment. Exclusion criteria included age under 2 years, the presence of a clear source of infection (e.g., otitis media, sinusitis, purulent pharyngitis, skin and soft tissue infection, urinary tract infection, pneumonia, dental abscess, osteomyelitis), or refusal to provide informed consent. Acute-phase samples consisted of whole blood and serum collected at the time of initial evaluation in the emergency department, within 14 days of symptom onset. Convalescent-phase samples were obtained approximately 2–3 weeks later. Samples were stored in the Laboratorio de Bacteriología Especial of the Pontificia Universidad Javeriana in Bogotá D.C., Colombia, until processing, and aliquots were subsequently shipped to the University of Texas Medical Branch in Galveston, TX, USA, for further analyses.

The study protocol and informed consent and/or assent forms were approved by the Research and Ethics Committee, Faculty of Sciences, Pontificia Universidad Javeriana on 17 August 2021. Each participant voluntarily signed the informed consent before sample collection, and each patient decided whether their samples could be used in future studies. For children aged ≥ 2 years and < 6 years, as well as for patients in critical condition, informed consent was obtained from their parents or legal guardians. For patients between 6 years and 18 years of age, written assent was obtained before the parents or legal guardians signed the informed consent form. All information was anonymized by assigning numeric codes to each participant. All procedures performed in the present study adhered to health research regulations as stated in the Resolution 8430 of 1993 of the Ministry of Health of Colombia and the principles of the Declaration of Helsinki for research involving human subjects.

### DNA extraction

DNA was extracted from 200 μL of whole-blood sample from each febrile patient using the DNeasy^®^ Blood and Tissue Kit (250) (Qiagen, Hilden, Germany) according to the manufacturer’s instructions. DNA concentration and purity were assessed spectrophotometrically with a NanoDrop 2000 (Thermo Scientific, Wilmington, DE, USA), and quality was further evaluated by conventional polymerase chain reaction (PCR) targeting a 289-base pair (bp) fragment of the β-actin encoding gene (*ACTB*) using the primers XAHR 17 (CGGAACCGCTCATTGCC) and XAHR 20 (ACCCACACTGTGCCCATCTA) following conditions reported elsewhere [25] to rule out the presence of inhibitors. Non-template (molecular-grade water) and positive controls (human DNA) were included in study sets. Amplicons were visualized by electrophoresis in 1% agarose gels stained with SYBR^™^ Safe DNA Gel Stain (Invitrogen, Waltham, MA, USA).

### Detection of *Bartonella* spp., *Borrelia* spp., and *C. burnetii*

The presence of *Bartonella* spp. DNA was assessed by a genus-specific qualitative real-time PCR (qPCR) targeting a 301-bp fragment of the *ssrA* (transfer-messenger RNA [mRNA]) gene using the primers ssrA-F (GCTATGGTAATAAATGGACAATGAAATAA) and ssrA-R (GCTTCTGTTGCCAGGTG) [26], and PowerUp^™^ SYBR^™^ Green Master Mix (Applied Biosystems, Austin, TX, USA). Each qPCR reaction was performed in a final volume of 10 µL, containing 5 µL of PowerUp^™^ SYBR^™^ Green Master Mix (2×), 0.2 µM of each primer, 2 µL of template DNA, and nuclease-free water to complete the final volume. A sample was considered positive when a successful amplification was achieved with a cycle threshold (Ct) value ≤ 40. Positive (*Bartonella quintana* DNA obtained from an antigen-coated slide (Focus Technologies, Cypress, CA, USA) and non-template (molecular-grade water) controls were included for all reactions. All samples with Ct values ≤ 40 were analyzed by melting curve analysis to verify amplification specificity. Positive samples exhibited a single, well-defined melting peak with a melting temperature (Tm) within ± 3 °C of the positive control. No additional peaks or nonspecific amplification patterns were observed. Furthermore, all qPCR-positive samples were subjected to Sanger sequencing of the amplified fragment, confirming *Bartonella*-specific sequences.

Detection of *Borrelia* spp. DNA was performed using a genus-specific qPCR with a fluorescent TaqMan probe targeting a 148-bp fragment of the 16S rRNA gene (*rrs*) using the primers Bor16S3F (AGCCTTTAAAGCTTCGCTTGTAG) and Bor16S3R (GCCTCCCGTAGGAGTCTGG) and the probe Bor16S3P (FAM-CCGGCCTGAGAGGGTGAACGG-BHQ1) following the protocol described previously [27]. All reactions were carried out using the Platinum™ Quantitative PCR SuperMix-UDG master mix (Thermo Fisher Scientific, Waltham, MA, USA). Reactions were carried out in a final volume of 10 µL, consisting of 5 µL of Platinum^™^ Quantitative PCR SuperMix-UDG (2×), 0.5 µM of each primer, 0.5 µM of TaqMan probe, 1 µL of DNA template, and nuclease-free water to reach the final reaction volume. Non-template (molecular-grade water) and positive (*Borrelia anserina* DNA kindly donated by Dr. Marcelo Bahia Labruna of the Universidade de São Paulo) controls were included in all study sets. Samples with a Ct value ≤ 40 were considered positive.

Detection of *C. burnetii* DNA was carried out using a species-specific qPCR with a fluorescent probe targeting a 154-bp fragment of the *C. burnetii* transposase (*IS1111*) encoding gene, using the primers FCoxB (CAAGAAACGTATCGCTGTGGC) and RCoxB (CACAGAGCCACCGTATGAATC), and the probe PCoxB (FAM-CCGAGTTCGAAACAATGAGGGCTG-TAMRA), as described elsewhere [28]. All reactions were performed using the Platinum™ Quantitative PCR SuperMix-UDG (Thermo Fisher Scientific, Waltham, MA, USA). Amplifications were performed in a total reaction volume of 10 µL, containing 5 µL of Platinum™ Quantitative PCR SuperMix-UDG (2×), 0.5 µM of each primer, 0.5 µM of probe, 1 µL of extracted DNA, and molecular-grade water to adjust the final volume. Negative (molecular-grade water) and positive (*C. burnetii* DNA, provided by Dr. Marcelo Bahia Labruna, Universidade de São Paulo) controls were included in each amplification procedure. Reactions yielding Ct values ≤ 40 were interpreted as positive.

### Identification of *Bartonella* spp.

To achieve species-level identification, samples that tested positive for *Bartonella* spp. by qPCR were further analyzed using conventional PCR with the same *ssrA* primers for sequencing purposes. Additionally, six complementary molecular targets were amplified by conventional PCR: a 739-bp fragment of the citrate synthase (*gltA*) encoding gene [29, 30], an 825-bp fragment of the RNA polymerase β subunit (*rpoB*) encoding gene [31], a 585-bp fragment of the riboflavin synthase (*ribC*) encoding gene [32], a 900-bp fragment of the cell division protein (*ftsZ*) encoding gene [33], a 793-bp fragment of the heat-shock chaperonin protein (*groEL*) encoding gene [34, 35], and a 549-bp fragment of the 16S-23S rRNA intergenic spacer region (*ITS*) encoding gene [36] (Table 1). The same positive and non-template controls used in the *ssrA* qPCR protocol were included in all amplification reactions. PCR products were visualized by electrophoresis on 2% agarose gels stained with SYBR^™^ Safe DNA Gel Stain (Invitrogen, Waltham, MA, USA). All obtained amplicons were purified using the Wizard^®^ DNA Clean-Up System kit (Promega, Madison, WI, USA) and subsequently bi-directionally sequenced.

**Table 1:** Primers and sequences of complementary target genes used for molecular characterization of *Bartonella* spp

Molecular marker	Primer name	Sequence
*gltA*	CS443-F	GCTATGTCTGCATTCTATCA
	BhCS.1137n-R	AATGCAAAAAGAACAGTAAACA
*rpoB*	1400-F	CGCATTGGCTTACTTCGTATG
	2300-R	GTAGACTGATTAGAACGCTG
*ribC*	BARTON-1	TAACCGATATTGGTTGTGTTGAAG
	BARTON-2	TAAAGCTAGAAAGTCTGGCAACATAACG
*ftsZ*	Bfp1	ATTAATCTGCAYCGGCCAGA
	Bfp2	ACVGADACACGAATAACACC
*groEL*	groEL-F	GGAAAAAGTGGGCAATGAAG
	groEL-R	TCCTTTAACGGTCAACGCATT
*ITS*	QHVE-1	TTCAGATGATGATCCCAAGC
	QHVE-3	AACATGTCTGAATATATCTTC

### Phylogenetic analyses

Forward and reverse sequences were assembled and edited using SnapGene^®^ Viewer 8.1 software (GSL Biotech LLC, Boston, MA, USA). Consensus sequences were compared against the NCBI GenBank database using the BLASTn program. Reference sequences for all recognized *Bartonella* species and *Candidatus* status were retrieved from the GenBank genetic sequence database.

Sequence alignments were generated using the ClustalW algorithm [37] implemented in the MEGA X software version 10.0.5 [38]. A maximum likelihood (ML) phylogenetic tree was constructed using the GTR + GAMMA + I evolutionary model selected as the best model on the basis of the Bayesian information criterion, with 1000 bootstrap replicates for branch support. The final ML phylogenetic tree was visualized and edited using iTOL version 5.0 [39].

### Serological detection of *Bartonella* spp. and *C. burnetti* infection

IgG antibodies against *Bartonella* spp. and *C. burnetii* were evaluated by indirect immunofluorescence assay (IFA). All convalescent-phase serum samples were initially tested, and when reactive, the corresponding acute-phase serum sample was also analyzed to assess seroconversion.

IFA was performed using commercial antigen-coated slides fixed with whole-cell antigens of *B. henselae* strain Houston-1 and phase I/II antigens of *C. burnetii* Nine Mile strain (Fuller Laboratories, Fullerton, CA, USA).

Samples were screened at a 1:128 dilution, and Alexa Fluor^®^ 488 goat anti-human IgG (H + L) (Invitrogen, Waltham, MA, USA) was used as secondary antibody at 1:2000 dilution. Slides were examined at 100× magnification under an Olympus BX61 fully motorized fluorescence microscope (Evident Scientific, Inc., Waltham, MA, USA). Reactive samples were further titrated, along with their paired acute-phase serum sample, by two-fold serial dilutions until disappearance of specific fluorescence compatible with bacterial morphology, establishing the sample endpoint titer.

A case was considered positive for recent *Bartonella* spp. infection when seroconversion was demonstrated, either by the absence of antibodies in the acute-phase sample and their presence in the convalescent-phase sample, or by a four-fold increase in antibody titer between paired serum samples. Samples not meeting these criteria but containing antibodies at a 1:128 cut-off were interpreted as evidence of previous exposure to *Bartonella* spp.

For *C. burnetii*, seropositivity was established at titers ≥ 1:128. Recent infection was defined by higher antibody titers against phase II antigens compared with phase I, or by a four-fold rise in phase II titers between acute and convalescent serum samples. Predominant reactivity to phase I antigens (≥ 1:128) was interpreted as indicative of chronic infection.

## Results

### Samples

A total of 56 patients were originally recruited from the studied region, of whom only 41 provided authorization for use of their samples in subsequent studies not originally included in the main research project. These 41 samples were utilized in the present study. DNA extraction from all whole-blood selected samples was successful, as evidenced by the amplification of the *ACTB* gene in 100% of them (41/41). The extracted DNA was subsequently used for the molecular detection of the bacterial agents evaluated in the present study.

### Detection of *Bartonella* spp., *Borrelia* spp., and *C. burnetii*

A total of 41 samples from febrile patients were screened by qPCR for the presence of *Bartonella* spp., *Borrelia* spp., and *C. burnetii*. Amplification of the *ssrA* gene revealed the presence of *Bartonella* spp. DNA in 9.8% (4/41) of the samples. Considering age, *Bartonella* qPCR-positive cases were observed only among children, young adults, and middle-aged adults. All positive cases were detected exclusively in male patients and originated solely from urban areas. Regarding the timing of sample collection, positive cases were recorded in September, November, and December. No amplification was observed for *Borrelia* spp. or *C. burnetii* in any of the samples tested, while all positive controls included in all reactions were amplified successfully.

### Identification of *Bartonella* spp.

Of the four samples positive for the *ssrA* gene by qPCR, all yielded an amplification product of the expected size by conventional PCR. Additional amplification of complementary genes was observed in two samples: One sample was positive for the *gltA* gene and another for the *ITS* gene. No amplification was obtained for any of the remaining complementary genes in the evaluated samples. Considering the total number of genes amplified per individual, two samples showed amplification of two genes, while the remaining two samples amplified a single gene. Of the six PCR-positive samples, all were sequenced bidirectionally. However, only four sequences showed sufficient quality and concordance between forward and reverse reads to generate reliable consensus sequences suitable for phylogenetic analysis, all corresponding to the *ssrA* gene from febrile patients.

**Fig. 1:**
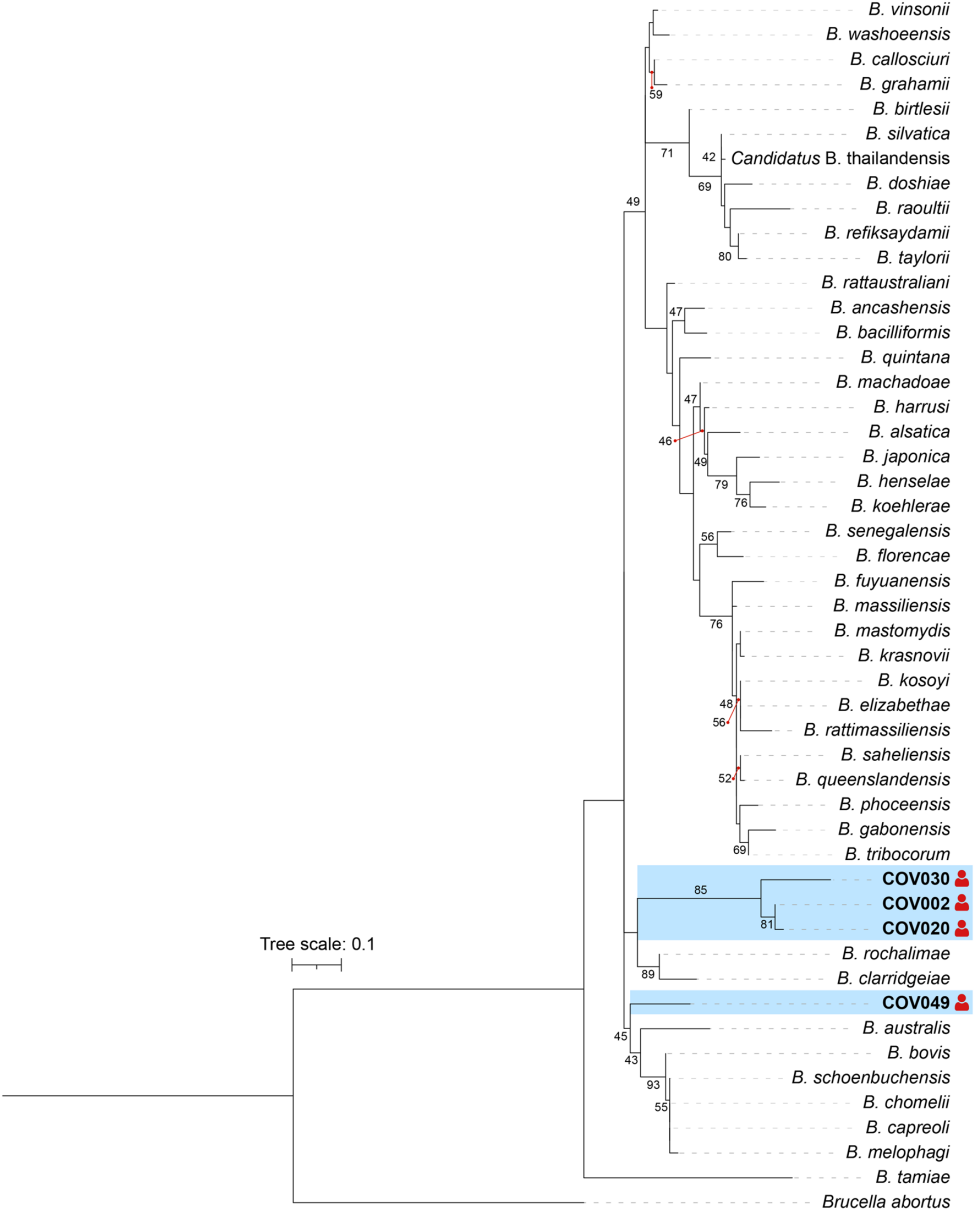
Maximum likelihood phylogenetic tree of *Bartonella* spp. based on *ssrA* sequences detected in febrile patients from Villeta municipality, Colombia. Sequences retrieved in the present study are signaled with human red icons and marked by a blue box. Only bootstrap values above 40% are shown

Phylogenetic analysis revealed that none of the good-quality sequences obtained in the present study could be classified to the species level. The sequences clustered together forming two distinct clades. The first clade included three sequences (COV002, COV020, and COV030), which were more closely related to *Bartonella clarridgeiae* and *Bartonella rochalimae*. The second clade included a single sequence (COV049), which exhibited a greater divergence, forming an independent branch most closely related to *Bartonella australis*, as well as to *Bartonella bovis*, *Bartonella schoenbuchensis*, *Bartonella chomelii*, *Bartonella melophagi*, and *Bartonella capreoli* (Fig. 1).

### Serological detection of *Bartonella* spp. and *Coxiella burnetii* infection

A total of 29.3% (12/41) of the samples screened showed the presence of antibodies against *Bartonella* spp., of which 9.8% (4/41) demonstrated seroconversion between the paired acute- and convalescent-phase serum samples, indicating recent exposure to *Bartonella* spp. Among the remaining samples, 19.5% (8/41) showed the presence of anti-*Bartonella* antibodies, but they did not exhibit seroconversion and were therefore classified solely as evidence of prior exposure.

Seropositive cases were identified in four age groups, including children, young adults, middle-aged adults, and older adults. Antibodies against *Bartonella* spp. were detected in both sexes. All seropositive individuals resided in urban areas and were identified between September and December.

Of the evaluated febrile patients, three demonstrated seroconversion, three were PCR-positive, and only one exhibited both seroconversion and PCR positivity, indicating concurrent molecular and serological evidence of infection. A detailed summary of all positive cases is presented in Table 2.

**Table 2:** Molecular and serological findings in febrile patients positive for *Bartonella* spp. from Villeta, Colombia, including demographic characteristics, PCR findings, IFA results, and IgG titers in acute- and convalescent-phase serum samples

Sample ID	Age group (years)	Sex	Origin	Recruitment month	Molecular screening result	Serological screening result	Acute-phase IgG titer	Convalescent-phase IgG titer
COV002	Child (8)	Male	Urban	September	Positive	Exposure	1:512	1:512
COV004	Middle-aged adult (31)	Male	Urban	September	Negative	Exposure	1:256	1:512
COV009	Child (12)	Male	Urban	October	Negative	Exposure	1:512	1:512
COV016	Older adult (81)	Male	Urban	November	Negative	Seroconversion	Negative	1:256
COV019	Young adult (18)	Male	Urban	November	Negative	Seroconversion	1:512	1:2048
COV020	Young adult (20)	Male	Urban	November	Positive	Negative	N/A	N/A
COV022	Young adult (19)	Female	Urban	November	Negative	Exposure	1:256	1:512
COV026	Young adult (26)	Male	Urban	December	Negative	Exposure	1:1024	1:1024
COV030	Middle-aged adult (33)	Male	Urban	December	Positive	Seroconversion	Negative	1:512
COV032	Middle-aged adult (34)	Female	Urban	December	Negative	Exposure	1:256	1:512
COV043	Older adult (59)	Female	Urban	December	Negative	Seroconversion	Negative	1:256
COV049	Middle-aged adult (44)	Male	Urban	December	Positive	Negative	N/A	N/A
COV052	Young adult (19)	Female	Urban	December	Negative	Exposure	1:128	1:256
COV056	Young adult (29)	Male	Urban	December	Negative	Exposure	1:128	1:128

No evidence of antibodies against either phase I or phase II antigens of *C. burnetii* was observed in any of the samples tested.

## Discussion

Villeta is an endemic area for several etiologies of AUFI, mainly SFG rickettsioses and leptospirosis [20, 21, 40]. However, the contribution of other neglected bacterial pathogens to the local AUFI burden remains poorly understood. In the present study, the detection of *Bartonella* spp. DNA and the demonstration of seroconversion in febrile patients from this region provided strong evidence of recent infection and suggested that *Bartonella* may be involved in AUFI cases within the studied cohort, although larger studies are required to clarify its epidemiological relevance in the region.

Serological screening revealed a seropositivity rate of 29.3% to *Bartonella* spp., a finding that is epidemiologically relevant given the recent emergence and increasing recognition of *Bartonella* spp. as a cause of human disease [41, 42]. Studies addressing *Bartonella* infection in the human population in Colombia remain scarce; however, a few studies have reported seropositivity rates ranging between 37.7% and 48.7% in rural and urban areas of Cordoba Department [43, 44], while a study conducted among the homeless population in Bogotá documented a seroprevalence of 19% [45]. These findings, together with data obtained in the present study, demonstrate that *Bartonella* circulates widely across different human populations and social contexts in Colombia, underscoring its importance as a potential public health concern in the country.

Additionally, molecular evidence of active *Bartonella* infection was obtained in 9.8% of febrile patients, with a similar proportion showing seroconversion. Notably, one patient tested positive by both molecular detection and seroconversion, reinforcing the potential etiological role of *Bartonella* in AUFI in the region. Although the role of *Bartonella* as a cause of AUFI in Colombia remains unclear, and this study represents the first attempt to investigate it, reports from other regions have demonstrated *Bartonella*’s involvement in febrile illnesses across different epidemiological contexts. A study in Malaysia showed that *Bartonella* infection is not uncommon among febrile patients [46]. Similarly, in several low- and middle-income African countries, *Bartonella* infection has been identified among patients with persistent fever [47]. Even more relevant, in Korea, recent *B. henselae* infections have been detected through seroconversion in patients with AUFI [48]; while in the Southwestern USA, cases of seroconversion to rodent-associated *Bartonella* antigens have been documented in febrile patients [49]. Similar findings were also reported in Thailand, where several *Bartonella* genotypes phylogenetically related to rodent-associated species were detected in the blood of febrile patients [50]. Collectively, this evidence supports that the occurrence of active and recent *Bartonella* infections in the present study are not isolated findings but rather part of a broader global pattern underscoring the zoonotic and clinical significance of *Bartonella* as a neglected etiology of AUFI globally.

It is important to acknowledge that serological testing was performed using commercial IFA slides coated with *B. henselae* antigens, whereas molecular analyses identified *Bartonella* DNA corresponding to genetically distinct lineages that could not be assigned to species level. Therefore, the serological findings cannot be directly attributed to the specific molecular lineages detected in this study. Cross-reactivity among *Bartonella* species has been well documented, particularly among phylogenetically related taxa (e.g., *B. henselae* and *B. quintana*), and may influence both the sensitivity and specificity of IFA-based assays. Consequently, seropositivity or seroconversion does not necessarily confirm infection with the same *Bartonella* lineage identified by molecular methods, but rather indicates exposure to antigenically related *Bartonella* organisms. This potential mismatch should be carefully considered when interpreting the combined serological and molecular results. Ideally, the use of a broader panel of species-specific antigens representing diverse lineages would allow a more accurate assessment of exposure patterns.

The *Bartonella* distribution according to demographic and biological variables revealed no relevant patterns, likely owing to limited sample size (*n* = 41). Nevertheless, it is noteworthy that all patients with active infection or serological evidence of exposure were exclusively from urban areas. Although *Bartonella* species exhibit wide diversity and a broad host range, several species such as *B. henselae*, *B. clarridgeiae*, *B. koehlerae*, and *B. vinsonii* subsp. *berkhoffii*, are commonly associated with domestic cats and dogs, as well as their fleas, particularly *Ctenocephalides felis*, in urban environments [51, 52]. Additionally, species such as *B. quintana* affect socially vulnerable urban populations [20, 45]. All these species have been associated with febrile illnesses of unknown origin. Therefore, it is possible that *Bartonella* species involved in AUFI in Villeta may be circulating predominantly within urban, domestic, or peri-domestic transmission cycles, involving companion animals and their ectoparasites. Although this hypothesis must be interpreted cautiously owing to the study’s limited sample size and scope, the small number of cases precludes robust inference regarding transmission patterns or urban predominance, and this observation should be further evaluated in larger, specifically designed epidemiological studies.

Molecular characterization of *Bartonella* from positive samples of febrile patients revealed two genetically distinct lineages, both showing considerable divergence that precluded precise species identification. Nevertheless, both clades showed close phylogenetic relationships with recognized *Bartonella* species. The first comprised three sequences that clustered near *B. clarridgeiae* and *B. rochalimae*. *Bartonella clarridgeiae* has been implicated in cat-scratch disease, endocarditis, and asymptomatic bacteremia, and exhibits a broad geographic distribution, including regions of Latin America [53–55]. Domestic cats and dogs serve as major sources of infection, and transmission occurs mainly by *C. felis* fleas [50, 53]. *B. rochalimae*, however, is known to cause fever of unknown origin, in addition to endocarditis and asymptomatic bacteremia in humans, and it has also been reported as a pathogen of domestic dogs [56–58]. This species is widely distributed, including in Latin America, and infects a wide range of domestic and wild mammals, as well as their associated flea ectoparasites [59, 60]. Although the sequences identified in this study were too divergent to confirm belonging to these species, their phylogenetic proximity suggests they could represent genetically related lineages, possibly sharing similar biological features such as human infectivity and flea-borne transmission. However, further studies are required to confirm this hypothesis and to clarify their transmission cycles.

The second lineage consisted of a single, most divergent sequence, most closely related to *B. australis*, *B. bovis*, *B. schoenbuchensis*, *B. chomelii*, *B. capreoli*, and *B. melophagi*. *Bartonella australis* appears to be restricted to Australia and surrounding regions, with no confirmed evidence of human infection [61]. The other species are typically associated with domestic and wild ruminants, such as cattle, sheep, and deer, in different parts of the world [50, 62, 63]. Although some have occasionally been detected infecting humans, solid evidence of pathogenicity is lacking [64, 65]. While the sequence obtained in the present study was too divergent to confirm identity with any of these species, its phylogenetic relatedness suggests potential biological similarities that warrant further investigation to elucidate its identity and possible relevance to human health.

Although the *Bartonella* sequences identified in the present study showed marked genetic divergence from previously described species, they may share conserved traits within the genus. Definitive taxonomic classification at the species level is not possible given that only a single marker, the *ssrA* gene, was obtained. Previous studies have shown that this molecular marker is highly sensitive for *Bartonella* detection and suitable for species-level differentiation compared with other genes [66, 67]. Nevertheless, considering the growing number of *Bartonella* species recognized in recent years, the use of multiple genetic markers or ideally bacterial isolation with whole-genome sequencing remains essential for more accurate characterization [66]. Amplification of additional genetic loci was largely unsuccessful. This limitation may be partially explained by low bacterial load in peripheral blood during acute *Bartonella* infection, as bacteremia levels are often low and intermittent, which can compromise the sensitivity of conventional PCR assays targeting longer or less sensitive loci. Reliance on a single genetic target restricts phylogenetic resolution and limits definitive taxonomic assignment; therefore, phylogenetic interpretations presented in this study should be considered preliminary. Future investigations incorporating multilocus sequence typing (MLST) or ideally bacterial isolation followed by whole-genome sequencing will be essential to achieve accurate species-level characterization and confirm the taxonomic status of the detected lineages.

Taken together, the results of the present study confirm the presence of two genetically distinct *Bartonella* lineages in febrile patients from Villeta municipality, neither corresponding to any previously described species but both related to taxa capable of infecting humans. These findings suggest the possible existence of previously undescribed *Bartonella* lineages with unrecognized zoonotic potential. However, confirmation of taxonomic status, pathogenicity, and epidemiological impact will require larger studies incorporating expanded sampling, culture, bacterial isolation, and multilocus or genomic analyses.

This study has some limitations. The relatively small sample size (*n* = 41) and restricted surveillance period limit the ability to draw robust epidemiological conclusions regarding prevalence, transmission patterns, or population-level distribution. The observed frequencies should therefore be interpreted as descriptive findings within this specific cohort rather than representative estimates for the broader population of Villeta. Additionally, the study design does not allow definitive causal attribution of *Bartonella* as the etiological agent of AUFI but rather supports its possible involvement in selected cases.

Regarding *Borrelia* spp. and *C. burnetii*, no positive results were obtained in the present study. Although historical reports suggested the presence of relapsing *Borrelia* in the region [22, 23], the absence of molecular detection in the present work may reflect limited sample size, current circulation, low bacterial load, or temporal fluctuations in transmission dynamics. To clarify this, specific serological studies targeting RFG *Borreliae* should be conducted in the future to determine whether these spirochetes currently circulate in the area. Finally, a previous investigation carried out in the same region also attempted to detect *C. burnetii* circulation without success [20], suggesting that its presence in local transmission cycles might be infrequent or sporadic. Consistent with those observations, the absence of both molecular and serological evidence in the present study supports the notion that if *C. burnetii* is circulating in Villeta, its occurrence is likely rare, limited to undetected transmission foci, or even not present in the region.

## Conclusions

This study provides the first molecular and serological evidence supporting the circulation of *Bartonella* spp. among febrile patients in Villeta, Colombia. Molecular analyses revealed two genetically distinct *Bartonella* lineages, both divergent from previously described species, suggesting the possible presence of previously undescribed lineages with potential zoonotic significance. The combined detection of bacterial DNA and serological reactivity, including confirmed seroconversion in several patients, indicates both active and past exposures, reinforcing the potential etiological role of *Bartonella* in AUFI in the region. The predominance of *Bartonella* infection among patients from urban areas should be interpreted cautiously given the limited cohort size and does not allow definitive conclusions regarding transmission dynamics; however, it raises the hypothesis of possible domestic or peri-domestic transmission cycles potentially involving companion animals and their ectoparasites, which should be explored in future studies. These findings underscore the value of a One Health approach in future studies, integrating human, animal, and environmental surveillance to fully elucidate transmission dynamics and zoonotic risk. Overall, these results highlight the importance of considering *Bartonella* in cases of AUFI in the region and emphasize the need for larger epidemiological investigations to determine its true prevalence, distribution, and public health impact.

## Data Availability

Data supporting the main conclusions of this study are included in the manuscript.

## Abbreviations

AUFI: Acute undifferentiated febrile illness
BLASTn: Basic Local Alignment Search Tool Nucleotide
bp: Base pair
Ct: Cycle threshold
DENV: Dengue virus
DNA: Deoxyribonucleic acid
IFA: Indirect immunofluorescence assay
IgG: Immunoglobulin G
H + L: Heavy and light chains
ML: Maximum likelihood
NCBI: National Center for Biotechnology Information
OROV: Oropouche virus
PCR: Polymerase chain reaction
qPCR: Quantitative PCR
RFG: Relapsing fever group
RNA: Ribonucleic acid
rRNA: Ribosomal RNA
SFG: Spotted fever group
